# Craniofacial Phenotypes and Genetics of DiGeorge Syndrome

**DOI:** 10.3390/jdb10020018

**Published:** 2022-05-13

**Authors:** Noriko Funato

**Affiliations:** Department of Signal Gene Regulation, Advanced Therapeutic Sciences, Medical and Dental Sciences, Graduate School of Medical and Dental Sciences, Tokyo Medical and Dental University (TMDU), Tokyo 113-8510, Japan; noriko-funato@umin.ac.jp

**Keywords:** 22q11.2 deletion syndrome, DiGeorge syndrome, velocardiofacial syndrome, cleft palate, skull base, cleidocranial dysplasia, hyoid bone, teeth abnormalities

## Abstract

The 22q11.2 deletion is one of the most common genetic microdeletions, affecting approximately 1 in 4000 live births in humans. A 1.5 to 2.5 Mb hemizygous deletion of chromosome 22q11.2 causes DiGeorge syndrome (DGS) and velocardiofacial syndrome (VCFS). DGS/VCFS are associated with prevalent cardiac malformations, thymic and parathyroid hypoplasia, and craniofacial defects. Patients with DGS/VCFS manifest craniofacial anomalies involving the cranium, cranial base, jaws, pharyngeal muscles, ear-nose-throat, palate, teeth, and cervical spine. Most craniofacial phenotypes of DGS/VCFS are caused by proximal 1.5 Mb microdeletions, resulting in a hemizygosity of coding genes, microRNAs, and long noncoding RNAs. *TBX1*, located on chromosome 22q11.21, encodes a T-box transcription factor and is a candidate gene for DGS/VCFS. TBX1 regulates the fate of progenitor cells in the cranial and pharyngeal apparatus during embryogenesis. *Tbx1*-null mice exhibit the most clinical features of DGS/VCFS, including craniofacial phenotypes. Despite the frequency of DGS/VCFS, there has been a limited review of the craniofacial phenotypes of DGC/VCFS. This review focuses on these phenotypes and summarizes the current understanding of the genetic factors that impact DGS/VCFS-related phenotypes. We also review DGS/VCFS mouse models that have been designed to better understand the pathogenic processes of DGS/VCFS.

## 1. Introduction

The 22q11.2 deletion syndrome is one of the most common chromosomal microdeletions, affecting approximately 1 in 4000 live births in humans [1]. A 1.5 to 2.5 Mb hemizygous deletion of chromosome 22q11.2 causes DiGeorge syndrome (DGS; OMIM #188400) and velocardiofacial syndrome (VCFS or Shprintzen VCF syndrome; OMIM #192430) [2]. DGS/VCFS appears to be a genomic disorder distinct from 22q11.2 distal deletion syndrome (OMIM #611867). The clinical phenotype of DGS/VCFS is a complex and variable congenital disability, including cardiovascular defects, thymic hypoplasia, parathyroid hypoplasia, and craniofacial malformations [3]. Craniofacial malformations occur in approximately 60% of patients with DGS/VCFS [4].

*TBX1*, located on chromosome 22q11.21, encodes a T-box transcription factor and is considered a candidate gene for DGS/VCFS since mutations in *TBX1* have been found in patients with DGS/VCFS [5]. Heterozygous *Tbx1*-mutant (*Tbx1*^+/−^) mice exhibit DGS/VCFS-related cardiovascular, parathyroid, and thymic phenotypes, suggesting that *TBX1* dosage is critical for cardiovascular, parathyroid and thymic development [6,7,8,9]. *Tbx1*-null mice exhibit the most clinical features of DGS/VCFS, including craniofacial phenotypes, while *Tbx1***^+/^**^−^ mice exhibit no significant craniofacial phenotypes [6,7,8,9,10].

There have been some excellent reviews on genetics and cardiovascular anomalies of DGS/VCFS [3,11,12,13]. However, information on the craniofacial anomalies of DGS/VCFS is limited. This review focuses on these phenotypes and summarizes the current understanding of the genetic factors that impact DGS/VCFS-related phenotypes. We also review DGS/VCFS mouse models that have been designed to better understand the pathogenic processes of DGS/VCFS.

## 2. Craniofacial Phenotypes of Patients with DGS/VCFS

Patients with DGS/VCFS manifest craniofacial anomalies involving the cranium, cranial base, jaws, pharyngeal muscles, ear-nose-throat, palate, teeth, and cervical spine (Figure S1, Table 1 and Table 2). Frequently observed craniofacial phenotypes include velopharyngeal insufficiency (27–92%), enamel hypomineralization (39–41%), hearing loss (33–39%), platybasia (50–91%), and cervical spine anomalies (75%) (Table 1). Delayed development of the hyoid bone has also been reported [14,15].

In addition to morphological anomalies, infants and young children with DGS/VCFS often exhibit a high prevalence of functional difficulties in feeding and speech/language associated with cleft palate, laryngeal anomalies, and velopharyngeal dysfunction [37]. Even after cleft palate closure, children with DGS/VCFS sometimes present communication disorders related to speech-language problems, such as articulation disorders of speech sounds and vocal disorders [37]. They exhibit slower language acquisition than those with other disorders that may be associated with abnormal muscle development.

## 3. Genetics of DGS/VCFS

**Figure 1:**
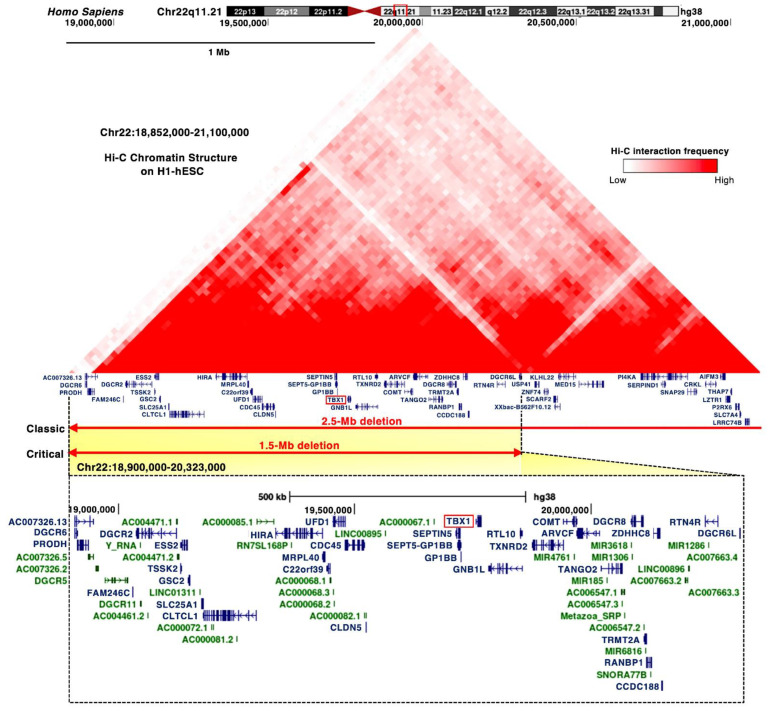
Proximal deletions of chromosome 22q11.2 are responsible for the clinical features of DGS/VCFS. Snapshot of the UCSC Genome Browser (http://genome.ucsc.edu accessed on 3 August 2021) in the hg38 assembly showing the genomic context in the proximal deletions of chromosome 22q11.2. Top, the 25 kb resolution Hi-C data in H1 human embryonic stem cell line (H1-hESC). Bottom, the coding (blue) and noncoding RNAs (green), including miRNAs and long noncoding RNAs, are shown.

DGS/VCFS is caused by a 1.5 to 2.5 Mb hemizygous deletion of chromosome 22q11.2 (Figure 1). Chromosomal microdeletions at 10p14-p13 (the *DGS2* locus) in patients with DGS/VCFS phenotypes are defined as the DGS/VCFS complex 2. In this review, we focus on the 22q11.2 locus, its associated genes, and miRNAs.

Most of the chromosomal deletions of the 22q11.2 locus are de novo, but inherited deletions of the 22q11.2 locus have been reported in 6–28% of patients as autosomal dominant [16,17]. The majority of clinical phenotypes of DGS/VCFS are caused by proximal 1.5 Mb microdeletions [3,22], resulting in a hemizygosity of approximately 30 coding genes, including *DGCR6, PRODH, DGCR2, ESS2, TSSK2, GSC2, FAM246C, SLC25A1, CLTCL1, UFD1, HIRA, CDC45, MRPL40, C22orf39, CLDN5, TBX1, SEPTIN5, SEPT5-GP1BB, GP1BB, GNB1L, RTL10, TXNRD2, COMT, ARVCF, TANGO2, TRMT2A, RANBP1, CCDC188, DGCR8, ZDHHC8, RTN4R, DGCR6L,* and *C007326*, as well as microRNAs (miRNAs) and long noncoding RNAs (Figure 1 and Figure S2A). The Hi-C chromatin structure of the 1.5 Mb region indicates interactions between these loci and their neighboring regions (Figure 1).

### 3.1. TBX1 Gene

The proximal deletion of 1.5 Mb on the 22q11.2 locus includes *TBX1* (Figure 1). *TBX1* is considered a candidate gene of DGS/VCFS because haploinsufficiency of *TBX1* leads to the typical phenotypes of DGS/VCFS, conotruncal anomaly face syndrome (OMIM #217095), and tetralogy of Fallot (OMIM #187500) (Table 3). Identical mutations in *TBX1* present among patients resulted in distinct phenotypes, suggesting that genetic and epigenetic changes or environmental factors are involved in the clinical phenotypes [5]. The coding variants in the T-box and C-terminal domains of TBX1 showed high combined annotation-dependent depletion (CADD) scores (Table S1); however, further investigation is required to confirm that the variants cause DGS/VCFS and how they impact the phenotypes.

### 3.2. DiGeorge Syndrome Critical Region (DGCR)

DGCR8, DGCR6, and DGCR6L map to the commonly deleted 1.5 Mb region in DGS/VCFS (Figure 1). DGCR8 is a nuclear miRNA-binding protein required for miRNA biogenesis. Dgcr8 haploinsufficiency in mice reduces the expression of miRNAs in the brain [45]. DGCR6 and DGCR6L genes encode a protein with a sequence similar to the Drosophila gonadal [46] (Figure S2B). In a chicken model, targeting DGCR6 function resulted in a vascular phenotype [47]. Attenuation of DGCR6 affects the expression of three genes localized within the 1.5 Mb region, upregulating the expression of TBX1 and UFD1 and reducing the expression of HIRA in the heart and pharyngeal arches of the chicken embryos [47]. Thus, the haploinsufficiency of DGCR8 or DGCR6 may be linked to DGS/VCFS phenotypes when targeting DGS/VCFS-related genes and miRNAs.

### 3.3. MicroRNAs

The deleted 1.5 Mb on the 22q11.2 locus includes several miRNAs, such as miR-185, miR-4716, miR-3618, miR-1286, miR-1306, and miR-6816 (Figure 1). The TargetScan miRNA target prediction program (http://www.targetscan.org accessed on 3 August 2021) identified that the 3′ UTR of *TBX1* includes conserved sites for miR-183-5p, miR-96-5p, miR-1271-5p, miR-182-5p, miR-144-3p, miR-139-5p, miR-101-3p, and miR-451. Two miRNAs were confirmed to target the 3′ UTR of *TBX1*. miR-96-5p represses *Tbx1* expression and, in turn, TBX1 suppresses the promoter activity and expression of miR-96 [48]. miR-451a, a tumor suppressor, also directly targets *TBX1* [49]. The expression of this gene is upregulated in cutaneous basal cell carcinoma, inversely to miR-451a [49]. miR-17-92 fine-tunes the expression of *Tbx1* in craniofacial development, suggesting miR-17-92 as a candidate genetic modifier for *Tbx1* [50]. Thus, miRNAs both inside and outside the 22q11.2 locus may influence the severity of the clinical phenotypes of DGS/VCFS.

## 4. Craniofacial Phenotypes of DGS/VCFS Mouse Models

**Figure 2:**
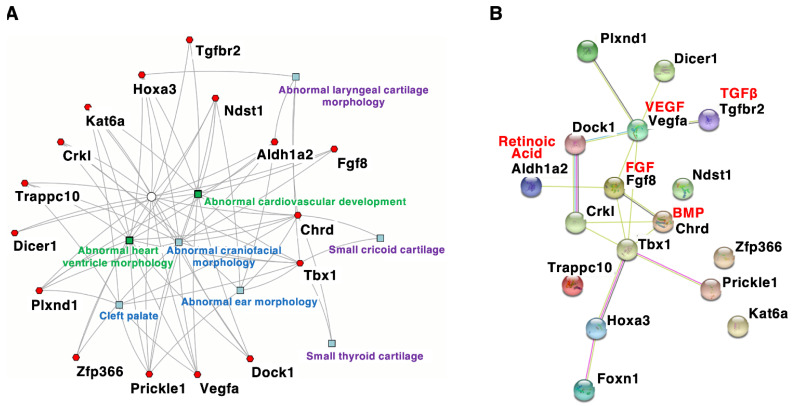
Interaction network of genes associated with DGS/VCFS phenotypes in mice. (**A**) A gene-based network where each gene connects to a feature. The network was constructed using ToppCluster (https://toppcluster.cchmc.org/ accessed on 6 May 2022). Mouse phenotypes are shown in the network. (**B**) The protein–protein interaction network was constructed using the STRING tool (https://string-db.org/ accessed on 6 May 2022). Genes associated with DGS/VCFS phenotypes in mice (Table 4) were the input. Different colors represent different types of evidence of a connection between proteins.

Mouse models with DGS/VCFS help identify additional candidate genes or modifier genes that influence the penetrance and/or severity of DGS/VCFS-related phenotypes. According to the mouse genome informatics (MGI) database (http://www.informatics.jax.org accessed on 3 August 2021), DGS/VCFS-related anomalies concerning Tbx1, Chrd, Tgfbr2, Vegfa, Fgf8, Crkl, Aldh1a2/Raldh2, Hoxa3, Kat6a/Moz/Myst3, Dicer1, Plxnd1, Dock1, Ndst1, Prickle1, Trappc10, Zfp366, and Foxn1 have been reported in genetically altered mice (Table 4 and Table S2). When these genes were analyzed according to biological process, “heart morphogenesis” and “cranial skeletal system development” were enriched (Table S3). Our enrichment analysis using ToppCluster [51] indicated that genes associated with DGS/VCFS phenotypes in mice are specifically enriched in the morphogenesis of craniofacial tissues and heart (Figure 2A). Interestingly, among these genes, only *Tbx1* and *Chrd* were specifically enriched in the morphogenesis of cricoid and thyroid cartilages (Figure 2A). Genes associated with DGS/VCFS phenotypes in mice also indicated that DGS/VCFS-related phenotypes involve the interaction of several signaling pathways, including bone morphogenetic protein (BMP), transforming growth factor (TGF)β, vascular endothelial growth factor (VEGF), fibroblast growth factor (FGF), and retinoic acid signaling pathways (Figure 2B). Genes involved in the genetic pathway of *Tbx1* are likely to induce phenotypes similar to *Tbx1*-null mice (Figure 2B, Table 4 and Table S2). These are described below.

### 4.1. Tbx1

Craniofacial structures with DGS/VCFS phenotypes are derivatives of the head mesenchyme and the first and second pharyngeal arches [62]. *Tbx1* is expressed in the mesoderm, ectoderm, and endoderm of the pharyngeal apparatus and head mesenchyme between embryonic day (E)9.5 and E11.5 in mice [62,63]. At E12.5, *Tbx1* is expressed in the oral epithelium, the myogenic core of the tongue, incisor tooth buds, pharyngeal muscles, and otic vesicle epithelium [63,64]. *Tbx1*-null mice exhibit the most clinical features of DGS/VCFS, while *Tbx1***^+/^**^−^ mice exhibit no significant craniofacial phenotypes (Table 2, Table 5 and Table S2). Information about ocular phenotypes in *Tbx1*-mutant mice is limited (Table 2), although these anomalies in patients with DGS/VCFS have been reported [16,17]. The Cre/loxP system has been used with *Tbx1* conditional knockout mice to examine the tissue-specific function of TBX1 in craniofacial development (Table 5).

#### 4.1.1. Cleft Palate

During palatogenesis, the palatal shelves develop bilaterally from the internal parts of the maxillary prominences and fuse above the tongue to form an intact oral cavity roof [67,68]. Because the palate consists of a bone-lined hard palate and a bone-free soft palate, cleft palate phenotypes include incomplete and submucosal cleft palates [67,68]. Ablation of *Tbx1*, which is expressed in the epithelium of the palatal shelves, results in abnormal intraoral epithelial fusions between the palatal shelves and the mandible, resulting in various degrees of the cleft palate phenotype (complete, incomplete, and submucosal cleft palate) [30,34,69]. Expression of *Pax9*, whose mutations lead to cleft palate and tooth agenesis [70], is downregulated in the palatal shelves and pharyngeal region of *Tbx1*-null embryos [34,71]. In *Tbx1*-null palatal shelves, muscle- and bone-related genes are downregulated, whereas neuron- and collagen biosynthesis-related genes are upregulated [72].

#### 4.1.2. Abnormalities in Craniofacial Bones

*Tbx1*-null mice display craniofacial bone abnormalities, including persistently open fontanelles, micrognathia, a short clavicle, a hypoplastic zygomatic arch, and the absence of the hyoid bone (Table 2 and Table S2). Conditional deletion of *Tbx1* in the mesoderm or osteochondral progenitors recapitulates the calvarial and mandibular phenotypes of *Tbx1*-null mice [35,66], suggesting that *Tbx1* is required for morphogenesis and ossification of craniofacial bones. Although *Tbx1* expression has not been reported in the neural crest, conditional deletion of *Tbx1* here results in a hypoplastic hyoid bone [35] (Table 2 and Table 5). These results indicate that *Tbx1* is required for the morphogenesis and ossification of mesoderm- and neural crest-derived membranous bones, although malformations observed in most neural crest-derived bones of *Tbx1*-null mice are secondary defects induced by non-neural crest cells [35,66]. Interestingly, abnormalities in membranous bones observed in *Tbx1*-null mice are similar to those of cleidocranial dysplasia (OMIM #119600 and #216330) in humans, exhibiting hypoplastic membranous bones, including abnormal neurocranial morphology, a short clavicle, a hypoplastic zygomatic arch, and hyoid bone [73,74,75]. Cleidocranial dysplasia (OMIM #119600) is caused by heterozygous mutations in *RUNX2*, which encodes a master transcription factor for osteoblast differentiation [74,75]. Since ablation of *Tbx1* affects *Runx2* expression in calvarial bones, and TBX1 overexpression induces *Runx2* expression in vitro [35], TBX1 may act upstream of *Runx2* by maintaining cell populations that express *Runx2* at the onset of bone development. In addition, *TBX1* could be a candidate gene for recessive inheritance of cleidocranial dysplasia (OMIM #216330).

#### 4.1.3. Abnormalities in the Cranial Base and Cervical Spine

The spheno-occipital synchondrosis (SOS) in the cranial base is a vital growth center for the skull (reviewed in [76]). TBX1 is expressed in the mesoderm-derived cartilage primordium of the SOS and basioccipital bones, and *Tbx1* deletion in the mesoderm induces malformed basioccipital bones and precocious ossified SOS. This indicates that *Tbx1* is an essential regulator of chondrocyte differentiation and subsequent ossification at the SOS [36]. TBX1 inhibits the transcriptional activity of RUNX2 in vitro as well as the expression of RUNX2 target genes in SOS [36]. *Tbx1*-null mice also exhibit endochondral bone abnormalities in the atlas, axis, and xiphoid process [6,35]. There is potential to examine the phenotypes of cranial synchondroses in DGS/VCFS patients, as abnormalities in the SOS and basioccipital bones may induce cranial phenotypes of DGS/VCFS, such as dolichocephaly, basilar impression, and platybasia.

#### 4.1.4. Dental Anomalies

Dental abnormalities (single central incisors, enamel hypoplasia, and small teeth) have been reported in many patients [18,28]. Accordingly, in approximately 30% of *Tbx1*-null mice, the upper incisors are absent [6]. *Tbx1* is expressed in the cervical loops, which contain the dental stem cell niche in mice. The cervical loop region of the incisor is either severely reduced or completely absent in *Tbx1*-null mice, and cultured incisors of *Tbx1*-null mice are hypoplastic and lack enamel [77]. Ablation of *Tbx1* in the epithelium results in smaller teeth than in the wild type, suggesting that TBX1 regulates the proliferation of dental progenitor cells [48].

#### 4.1.5. Muscle Hypotonia

Branchiomeric muscles are derived from the mesoderm of the pharyngeal arch. In *Tbx1*-null and *Tbx1*^flox/-^;*Mesp1*-*Cre* embryos, the masseter, pterygoid, and temporalis muscles are intermittently absent [78,79]. Accordingly, muscle-related genes are also downregulated in *Tbx1*-null palatal shelves [72]. *Tbx1* acts upstream of critical transcription factors to form branchiomeric muscles. These include LIM homeobox protein 2 (*Lhx2*), transcription factor 21 (*Tcf21/capsulin*), musculin (*Msc*), myogenic factor 5 (*Myf5*), myogenic differentiation 1 (*Myod1*), myocyte enhancer factor 2C (*Mef2c*), and GATA binding protein 4 (*Gata4*) [79,80,81,82]. *Tbx1* is in the downstream genetic pathways of *Tcf21*, paired-like homeodomain transcription factor 2 (*Pitx2*), and ISL LIM homeobox 1 (*Isl1*) [80,83,84]. Thus, TBX1 regulates the pattern and development of branchiomeric muscles through the transcriptional regulation of myogenic genes.

### 4.2. Chordin (Chrd) and Transforming Growth Factor, Beta Receptor II (Tgfbr2)

Mice lacking the *Chrd* gene encoding chordin, an antagonist of bone morphogenetic proteins (BMPs), exhibit recapitulating phenotypes in *Tbx1*-null mice [32,52] (Table S2). *Chrd*-null neonates exhibit most craniofacial phenotypes in the cranium, cranial base, maxilla, mandible, ears, and hyoid bone (Table S2). Both *Tbx1* and *Fgf8* were reduced in the endoderm of *Chrd*-null mice, indicating that *Chrd* acts upstream of *Tbx1* and *Fgf8* [52]. *Tbx1* acts upstream of SMAD family member 7 (*Smad7)*, an inhibitory Smad within the BMP/TGFβ pathway, to regulate vascular smooth muscle and extracellular matrix investment of the fourth arch artery [85]. Conditional deletion of *Tgfbr2*, which encodes TGFβ receptor 2, in the neural crest resulted in DGS/VCFS-related cardiovascular defects [53]. These findings suggest a potential role of BMP/TGFβ signaling in the pathogenesis of DGS/VCFS.

### 4.3. Vascular Endothelial Growth Factor A (Vegfa)

VEGFA is an essential cytokine in angiogenesis and vascular development during embryogenesis [86]. *Vegfa*-null neonates exhibit a few aspects of DGS/VCFS-related craniofacial anomalies, including unfused cranial sutures, absent incisors, and short mandibles, as well as cardiovascular abnormalities [54] (Table S2). The deletion of *Vegfa* in mice reduces *Tbx1* expression, and the knockdown of *vegfaa/vegfa* levels in zebrafish enhances the pharyngeal arch malformations induced by *tbx1* knockdown [54]. In humans, low expression of the *VEGFA* haplotype increases the risk of a cardiac phenotype of DGS/VCFS, indicating that expression levels of *VEGFA* affect the severity of DGS/VCFS phenotypes [87]. These results suggest that VEGFA modifies DGS/VCFS-related phenotypes by regulating *TBX1* expression.

### 4.4. Fibroblast Growth Factor 8 (Fgf8) and FGF Receptor 2 (Fgfr2)

Ablation of *Fgf8* induces craniofacial, cardiovascular, thymic, and parathyroid phenotypes [55,88]. *Fgf8*-null neonates exhibit a few aspects of DGS/VCFS-related craniofacial anomalies, including cleft palate and abnormal outer ear morphology [55,88] (Table S2). *Fgf8*^+/−^;*Tbx1*^+/−^ double heterozygous embryos show an increased penetrance of cardiovascular defects compared with *Tbx1*-heterozygous embryos [89]. Tissue-specific deletion of *Fgf8* in *Tbx1*-expressing domains results in cardiovascular anomalies [90]. TBX1 activates the *Fgf8* enhancer during cardiac development [9]. Deletion of the *Fgfr2* gene that encodes FGF receptor 2 decreases *Tbx1* expression in the dental epithelium, indicating a genetic link between FGF signaling and *Tbx1* in tooth development [91]. In addition, a *Tbx1-Six1/Eya1-Fgf8* genetic pathway is crucial for craniofacial morphogenesis [92,93]. These findings demonstrate that the FGF pathway and *Tbx1* interact genetically during pharyngeal arch development.

### 4.5. CRK like Proto-Oncogene, Adaptor Protein (Crkl)

*CRKL* maps to the 2.5 Mb region commonly deleted in DGS/VCFS (Figure 1). Variants in a predicted enhancer of *CRKL* are significantly associated with the risk of congenital heart defects in DGS/VCFS [94]. Approximately 12% of *Crkl*-null mice show mild cranial bone defects, such as small cranium and poor membranous ossification of the nasal bones [56]. Compound heterozygosity of *Crkl* and *Tbx1* in mice has revealed that *Crkl* deletion enhances DGS/VCFS-related abnormalities compared with *Tbx1*-heterozygous embryos [56], suggesting that *Tbx1* and *Crkl* genes act in the same genetic pathway. *CRKL* encodes an adaptor protein that promotes the intracellular response of FGF signaling. *Crkl*^+/−^;*Fgf8*^+/−^ double heterozygous mice showed DGS/VCFS-related defects [95]. Thus, *CRKL* mutations cause or modify DGS/VCFS-related phenotypes and/or penetrance as a contiguous gene syndrome.

### 4.6. Aldehyde Dehydrogenase Family 1, Subfamily A2 (Aldh1a2/Raldh2)

Retinoic acid (RA), an active vitamin A derivative, is essential for various developmental processes in vertebrates. High levels of RA act as morphogens that cause phenocopies of DGS/VCFS by downregulating *Tbx1* expression in the pharyngeal apparatus [96,97]. RA levels are balanced by the RA-synthesizing enzyme aldehyde dehydrogenase (ALDH) and the Cyp26 RA-catabolizing enzyme [98,99]. Mouse embryos hypomorphic for *Aldh1a2/Raldh2* display DGS/VCFS-related cardiovascular, thymic, and parathyroid malformations [57]. Haploinsufficiency of *Aldh1a2/Raldh2* results in reduced embryonic synthesis of RA, increased levels of *Tbx1*, and accelerated recovery from arterial growth delay in *Tbx1*-heterozygous mice [100]. An inhibitor of the Cyp26 enzyme induces a phenocopy of DGS/VCFS in chick embryos [101]. In *Tbx1*-null mice, upregulated expression of *Aldh1a2/Raldh2* and downregulated expression of *Cyp26a1* have been observed [71].

Further interactions occur between RA signaling, *Crkl*, and *Tbx1*. The penetrance of thymic hypoplasia is reduced in *Crkl^+/^*^−^;*Tbx1^+/^*^−^;*Aldh1a2^+/^*^−^ triple heterozygous embryos compared to *Crkl*^+/−^;*Tbx1*^+/−^ mutants, suggesting that reducing the amount of RA may rescue the DGS/VCFS-related phenotype [102]. Thus, the levels of RA in embryogenesis could contribute to the phenotypic variability of DGS/VCFS.

### 4.7. Homeobox A3 (Hoxa3)

RA exposure increases the expression of *Hoxa3*, a gene which encodes a homeobox transcription factor, in the neural tube and pharyngeal apparatus [103]. Interestingly, *Hoxa3*-null neonates show some aspects of the abnormalities of DGS/VCFS [58,104] (Table S2). Thus, *HOXA3* may be a genetic modifier of DGS/VCFS-related abnormalities.

### 4.8. Kat6a/Moz/Myst3 (Lysine Acetyltransferase 6A) and Epigenetic Modifiers

Homozygous mutation of *Kat6a/Moz/Myst3*, which encodes a histone acetyltransferase, leads to cardiovascular defects seen in DGS/VCFS and reduces *Tbx1* expression [59]. Treatment of pregnant mice with a histone demethylase inhibitor reportedly increased the methylation levels of histone H3 lysine K4 (H3K4) and partially rescued the cardiovascular phenotypes of *Tbx1*-heterozygous mice [105]. TBX1 regulates genes transcribed at a low level by recruiting lysine methyltransferase (KMT2C) and controlling monomethylation of H3K4 (H3K4me1) enrichment on chromatin [105]. In addition, TBX1 transcriptionally targets *Wnt5a* by interacting with SMARCD1/BAF60a, a component of the SWI/SNF-like BAF chromatin remodeling complex, along with the H3K4 monomethyltransferase SETD7 [106]. Microduplication in *KANSL1*, which encodes a member of the histone acetyltransferase complex, is associated with heart anomalies in individuals with DGS/VCFS [107]. In T cells of patients with DGS/VCFS, the status of transcriptional activation (H3K4me3 and H3K27ac) is globally increased [108]. Thus, epigenetic changes are involved in DGS/VCFS-related phenotypes.

### 4.9. Sonic Hedgehog (Shh)

*Shh* encodes an SHH signaling molecule. In humans, *SHH* mutations lead to holoprosencephaly 3 (OMIM #142945), microphthalmia with coloboma (OMIM #611638), and single median maxillary central incisor (OMIM #147250). *Shh*-null embryos exhibit conotruncal and pharyngeal arch artery defects similar to those observed in DGS/VCFS and *Tbx1*-null embryos [109]. *Tbx1* expression is reduced in *Shh*-null embryos, and ectopic expression of *Shh* can result in the upregulation of *Tbx1*, suggesting that *Shh* is a possible modifier for DGS/VCFS [62,110]. *Shh* is also required for the expression of the Fox family of transcription factor genes, forkhead box A2 *(Foxa2*) and forkhead box C2 *(Foxc2*), in the head mesenchyme and the pharyngeal endoderm [62]. FOXA2 and FOXC2 bind to regulatory regions in the mouse and human *TBX1* loci [111].

### 4.10. Paired-like Homeodomain Transcription Factor 2 (Pitx2)

*Pitx2* gene encodes a bicoid-like homeodomain transcription factor. *Pitx2*-null mice show craniofacial defects, such as the arrest of tooth development, abnormal morphology of maxilla and mandible, and cleft palate. In humans, *PITX2* mutations lead to Axenfeld–Rieger syndrome, type 1 (OMIM #180500). Patients with Axenfeld–Rieger syndrome manifest dental and craniofacial anomalies involving the maxilla, mandible, and cranial base [112]. Both *Tbx1* and *Pitx2* are expressed in the early dental epithelium, oral epithelium, and secondary heart field [64,113,114]. *Tbx1*^+/−^;*Pitx2*^+/−^ double heterozygous embryos exhibit increased penetrance of an extra premolar-like tooth [115] and DGS/VCFS-related cardiovascular anomalies [114]. TBX1 directly activates the *Pitx2c* enhancer through the synergistic action of the homeobox-containing transcription factor NK2 homeobox 5 (NKX2-5) [114]. TBX1 also interacts with PITX2 and represses PITX2 transcriptional activity [48,115]. Thus, *PITX2* may be a genetic modifier of DGS/VCFS-related abnormalities.

## 5. Discussion

The penetrance and severity of congenital anomalies are related to genetic and environmental factors. Recent studies have revealed the function of TBX1 and modifiers that impact the severity and penetrance of DGS/VCFS. Studies of DGS/VCFS mouse models have provided insights into signaling pathways and genes that interact with TBX1 and/or affect the DGS/VCFS phenotypes. In addition, mouse models with DGS/VCFS may help us to identify additional DGS/VCFS-related phenotypes. For example, there is potential to examine the phenotypes of cranial synchondroses, cranium, zygomatic arches, and pharyngeal muscles in DGS/VCFS patients. We also noted that information about ocular phenotypes in *Tbx1*-mutant mice is limited, although these anomalies in patients with DGS/VCFS have been reported [16,17]. Crosstalk with key embryonic signals, especially BMP, TGFβ, VEGFA, FGF, RA, and SHH, critically regulates DGS/VCFS-related pharyngeal development. Genes involved in these signaling pathways may modify the phenotypic spectrum of DGS/VCFS. Given the broad spectrum of DGS/VCFS disease phenotypes, other genes essential to craniofacial development could modify the phenotypic spectrum. Genetically engineered mice are useful for studying disease phenotypes; however, ablation of essential genes involved in cardiovascular development may cause early embryonic lethality, which would prevent observation of craniofacial phenotypes. For example, ablation of *Ufd1*, whose human ortholog has been mapped to the 1.5 Mb region, causes early embryonic lethality before organogenesis in mice [116]. It is also essential to identify novel proteins that interact with TBX1 and examine whether interacting partners may influence the phenotypes of mouse models.

## 6. Conclusions

Studies of *Tbx1*-mutant mice have provided insights into the underlying pathogenesis of DGS/VCFS and the knowledge to diagnose patients with DGS/VCFS. Genes, miRNAs, and epigenetics could change *Tbx1* expression. Polymorphisms, variations, and mutations in *TBX1* may induce the penetrance and severity of DGS/VCFS-like craniofacial phenotypes. The molecular basis of the variant sequence of *TBX1* will further define how *TBX1* contributes to the craniofacial and other phenotypes of DGS/VCFS. Since interactions with TBX1 and other molecules in transcriptional complexes or chromatin remodeling are crucial for TBX1 function, identifying and understanding these genetic and epigenetic modifiers individually for each patient may direct therapeutics to minimize the severity.

## Figures and Tables

**Table 1:** Craniofacial anomalies in patients with DGS/VCFS.

Phenotypes	Features	Frequency
Palatal anomalies	Overt cleft palate	7–11%
	Submucous cleft palate	5–23%
	Bifid uvula	5–10%
	Velopharyngeal insufficiency	27–92%
Dental anomalies	Tooth agenesis	15%
	Hypoplasia of primary teeth	32%
	Hypoplasia of permanent teeth	10%
	Enamel hypomineralization of primary teeth	39%
	Enamel hypomineralization of permanent teeth	41%
Ear-nose-throat abnormalities	Hearing loss	33–39%
	Otitis media with effusion	2%
	Tracheomalacia/laryngomalacia	2%
	Laryngeal web	1%
Ocular abnormalities	Hooding of the upper lid	41%
	Ptosis	9%
	Hooding of the lower lid	6%
	Epicanthal folds	3%
	Distichiasis	3%
Cranial base anomalies	Platybasia	50–91%
	Basilar impression	3%
Cervical spine anomalies	Atlas (C1) anomalies	75%
	Axis (C2) anomalies	59%
	Fusion of C2–C3	34%

Data were summarized from the following references: [16,17,18,19,20,21,22].

**Table 2:** Craniofacial and skeletal phenotypes of DGS/VCFS and *Tbx1*-null mice.

	DGS/VCFS	*Tbx1*-Null Mice
Cranium	Dolichocephaly	Small cranium
	Abnormal skull morphology	Hypoplastic parietal bone
	Malar flattening	Hypoplastic interparietal bone
	Long face	Unfused cranial sutures between frontal and parietal bones
		Temporal bone hypoplasia
		Absent zygomatic arch
		Abnormal zygomatic arch morphology
Cranial Base	Platybasia	Abnormal fusion of the basioccipital and basisphenoid bones
	Basilar impression	Abnormal presphenoid bone morphology
		Abnormal basioccipital bone morphology
Palate	Cleft palate	Cleft palate
	Submucous cleft palate	Submucous cleft palate
	Bifid uvula	Bifid uvula
	Highly arched palate	
	Velopharyngeal insufficiency	
Mandible	Retrognathia	Absent mandibular coronoid process
	Short mandible	Short mandible
	Micrognathia	Micrognathia
Teeth	Enamel hypoplasia	Abnormal upper incisor morphology
	Single central incisor	Absent upper incisors
	Small teeth	
	Abnormality of the dentition	
	Carious teeth	
Muscles	Pharyngeal hypotonia	Absent masseter muscle
		Absent pterygoid muscle
		Absent temporalis muscle
Eyes	Hypertelorism/telecanthus	Hypertelorism
	Downslanted palpebral fissures	
	Proptosis	
	Strabismus	
	Abnormal eyelid morphology	
	Epicanthus	
	Microphthalmia	
External Ears	Small earlobe	Ear lobe hypoplasia
	Low-set ears	Lowered ear position
	Abnormally folded pinna	Abnormal ear shape
	Preauricular pit	Absent outer ear
		Anotia
Middle and Inner Ears	Chronic otitis media	Abnormal middle ear ossicle morphology
	Conductive hearing loss	Absent middle ear ossicles
	Sensorineural hearing loss	Abnormal stapes morphology
	Auditory canal stenosis	Abnormal incus morphology
	Pulsatile tympanic membrane	Abnormal malleus morphology
	Thickened tympanic membrane	Absent stapes
	Tympanic membrane retraction	Abnormal external auditory canal morphology
		Decreased tympanic ring size
Nose	Prominent nasal bridge	Short snout
	Abnormal nasal morphology	
	Underdeveloped nasal alae	
	Choanal atresia	
Throat	Abnormal thorax morphology	Small thyroid cartilage
	Abnormality of the pharynx	Small cricoid cartilage
		Abnormal thyroid cartilage morphology
		Pharynx hypoplasia
Hyoid bones	Delayed development of the hyoid bone	Hyoid bone hypoplasia
	Invisible hyoid ossification center	Abnormal hyoid bone morphology
Cervical spine	Dysmorphic C1	Abnormal cervical atlas (C1) morphology
	Anterior arch cleft of C1	Absent arcus anterior of C1
	Open posterior arch C1	
	Fusion of C1–C2	
	Fusion of C2–C3	
	Upswept C2 lamina	
	Platyspondyly	
Others		Short clavicle
References	[14,15,16,17,18,19,20,21,22,23,24,25,26,27,28]	[6,7,8,9,10,29,30,31,32,33,34,35,36]

Data were summarized from the following references [6,7,8,9,10,14,15,16,17,18,19,20,21,22,23,24,25,26,27,28,29,30,31,32,33,34,35,36], OMIM (https://www.omim.org accessed on 3 August 2021) and the Monarch Initiative (https://monarchinitiative.org accessed on 3 August 2021).

**Table 3:** DGS/VCFS-associated variants of *TBX1.*

Mutation	Domain	Condition	Craniofacial Anomalies	References
c.89_284del	N-terminal	DiGeorge syndrome	Yes	ClinVar Variant: 971780
c.199_224del	N-terminal	DiGeorge syndrome	Yes	ClinVar Variant: 949172
c.292A>T	N-terminal	DiGeorge syndrome	Yes	ClinVar Variant: 526036
c.385G>A	T-box	Tetralogy of Fallot	No	ClinVar Variant: 488618
c.443T>A (F148Y)	T-box	Conotruncal anomaly face syndrome	Yes	[5]
c.503T>C	T-box	DiGeorge syndrome Velocardiofacial syndrome (Shprintzen syndrome) Tetralogy of Fallot	Yes	ClinVar Variant: 973222
c.569C > A (P190Q)	T-box	Congenital heart defects	No	[38]
c.582C>G (H194Q)	T-box	Velocardiofacial syndrome	Yes	[39]
c.928G>A (G310S)	C-terminal	DiGeorge syndrome	Yes	[5]
c.967_977dup AACCCCGTGGC	C-terminal	Thymic hypoplasia Postaxial polydactyly of the right fifth toe	No	[40]
c.1158_1159delinsT	C-terminal	Hypoparathyroidism and hypocalcemia Facial asymmetry Deafness	Yes	[41]
c.1223delC	C-terminal	Conotruncal anomaly face syndrome Velocardiofacial syndrome	Yes	[5]
c.1253delA	C-terminal	DiGeorge syndrome	Yes	[42]
c.1320-1342del23bp	C-terminal	Velocardiofacial syndrome	Yes/No	[43]
c.1399-1428dup30	C-terminal	Tetralogy of Fallot Scoliosis Facial asymmetry Upslanting palpebral fissures Absent pulmonary valve Isolated left pulmonary artery	Yes	[44]

ClinVar (https://www.ncbi.nlm.nih.gov/clinvar accessed on 3 August 2021).

**Table 4:** Craniofacial phenotypes of DGS/VCFS mouse model.

Gene Symbol	Induced Mutation Type	Cranium	Palate	Teeth	Muscles	Ear-Nose-Throat	Hyoid Bones	Cardio-Vascular
*Tbx1*	Null	Yes	Yes	Yes	Yes	Yes	Yes	Yes
*Chrd*	Null	Yes	Yes	nr	nr	Yes	Yes	Yes
*Tgfbr2*	Deletion (*Wnt1-Cre*)	Yes	Yes	nr	nr	nr	nr	Yes
*Vegfa*	Null	Yes	Yes	Yes	nr	nr	nr	Yes
*Fgf8*	Hypomorphic allele	Yes	Yes	Yes	nr	Yes	Yes	Yes
*Crkl*	Null	Yes	nr	nr	nr	Yes	nr	Yes
*Aldh1a2*	Hypomorphic allele	nr	nr	nr	nr	Yes	Yes	Yes
*Hoxa3*	Null	nr	Yes	nr	Yes	Yes	Yes	Yes
*Kat6a*	Null	nr	Yes	nr	nr	Yes	nr	Yes
*Dicer1*	Deletion (*Wnt1-Cre*)	Yes	nr	nr	nr	nr	nr	Yes
*Plxnd1*	Single point mutation	nr	Yes	nr	nr	Yes	nr	Yes
*Dock1*	Undefined	nr	nr	nr	nr	Yes	nr	Yes
*Ndst1*	Single point mutation	nr	nr	nr	nr	Yes	nr	Yes
*Prickle1*	Single point mutation	Yes	Yes	nr	nr	Yes	nr	Yes
*Trappc10*	Undefined	Yes	Yes	nr	nr	nr	nr	Yes
*Zfp366*	Single point mutation	nr	nr	nr	nr	Yes	nr	Yes
*Foxn1*	Intragenic deletion	nr	nr	nr	nr	Yes	nr	Yes

Mouse models of DiGeorge syndrome with phenotypic similarity to human diseases can be found in the Mouse Genome Informatics (MGI) database (http://www.informatics.jax.org accessed on 3 August 2021). Data were summarized from the following references [6,7,8,9,10,29,30,31,32,33,34,35,36,52,53,54,55,56,57,58,59,60,61]. nr, not reported. A detailed description is provided in Table S2.

**Table 5:** Selected craniofacial phenotypes of *Tbx1*-mutant neonates.

*Tbx1*-Mutant Mice		Craniofacial Phenotypes					
Mutation Type	Tissue/Cell	Cranium	Cranial Base	Palate	Mandible	Hyoid Bone	Cervical Spine
*Tbx1* ^+/−^	Entire body	Normal	Normal	Normal	Normal	Normal	Normal
*Tbx1*-null	Entire body	Abnormal	Abnormal	CP	Hypoplastic	Hypoplastic	Abnormal
Deletion (*Foxg1-Cre*)	Pharyngeal tissues *	Abnormal	Abnormal	CP	Hypoplastic	Hypoplastic	NA
Deletion (*KRT14-Cre*)	Epithelium	Normal	Normal	Anterior CP	Normal	Normal	Normal
Deletion (*Mesp1-Cre*)	Mesoderm	Abnormal	Abnormal	NA	Hypoplastic	Hypoplastic	Abnormal
Deletion (*Twist2-Cre*)	Osteochondral progenitors	Abnormal	Abnormal	Normal	Normal	Hypoplastic	Abnormal
Deletion (*Wnt1-Cre*)	Neural crest	Normal	Normal	Normal	Normal	Hypoplastic	Normal

Data were summarized from the following references: [30,31,34,35,36,65,66]. * pharyngeal pouches, otic and optic vesicles [30,31]; * pharyngeal endoderm, ectoderm, and mesoderm [65]; CP, cleft palate; NA, not available.

## Supplementary Materials

The following are available online at https://www.mdpi.com/article/10.3390/jdb10020018/s1, Figure S1: Craniofacial and skeletal phenotypes of DGS/VCFS. Figure S2: Human genes in the proximal deletion of 1.5 Mb on the 22q11.2 locus. Table S1: Craniofacial and skeletal phenotypes of DGS/VCFS and Tbx1-null mice. Table S2: Craniofacial and skeletal phenotypes in mouse models of DGS/VCFS. Table S3: Classification of mouse genes associated with DGS/VCFS.
