# Supplementary material for: Craniofacial Phenotypes and Genetics of DiGeorge Syndrome
Source: J Dev Biol. 2022 May 13;10(2):18. doi: 10.3390/jdb10020018 (PMC9149807; doi:10.3390/jdb10020018)
Supplement: Supplementary file 1 [file jdb-10-00018-s001.zip › jdb-1714769-supplementary Figures.pdf]

Figure S1

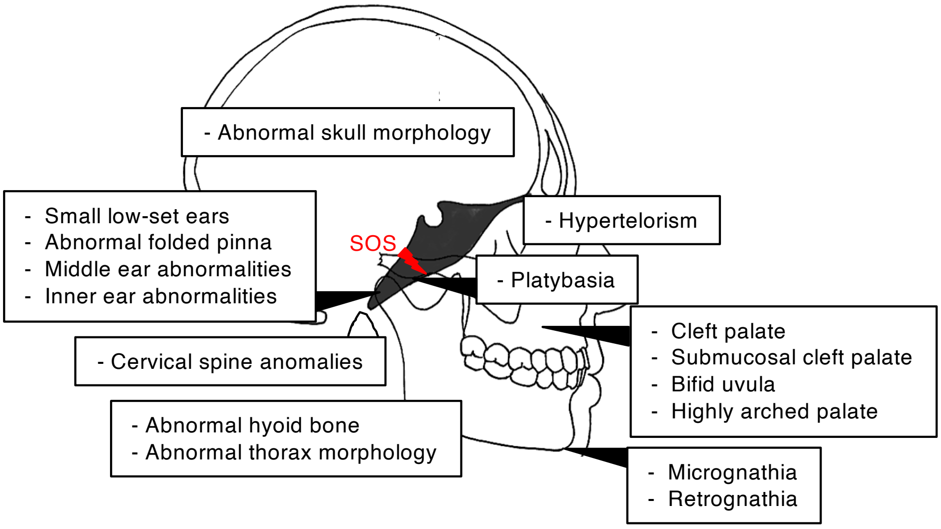

Figure S1. Craniofacial and skeletal phenotypes of DGS/VCFS.
SOS, spheno-occipital synchondrosis.

Figure S2

A

Symbol	Description	Gene ID
DGCR6	DiGeorge syndrome critical region gene 6	8214
PRODH	proline dehydrogenase 1	5625
DGCR2	DiGeorge syndrome critical region gene 2	9993
ESS2	ess-2 splicing factor homolog	8220
TSSK2	testis specific serine kinase 2	23617
GSC2	goosecoid homeobox 2	2928
FAM246C	family with sequence similarity 246 member C	117134596
SLC25A1	solute carrier family 25 member 1	6576
CLTCL1	clathrin heavy chain like 1	8218
UFD1	ubiquitin recognition factor in ER associated degradation 1	7353
HIRA	histone cell cycle regulator	7290
CDC45	cell division cycle 45	8318
MRPL40	mitochondrial ribosomal protein L40	64976
C22orf39	chromosome 22 open reading frame 39	128977
CLDN5	claudin 5	7122
TBX1	T-box transcription factor 1	6899
SEPTIN5	septin 5	5413
SEPT5-GP1BB	SEPT5-GP1BB readthrough	100526833
GP1BB	glycoprotein Ib platelet subunit beta	2812
GNB1L	G protein subunit beta 1 like	54584
RTL10	retrotransposon Gag like 10	79680
TXNRD2	thioredoxin reductase 2	10587
COMT	catechol-O-methyltransferase	1312
ARVCF	ARVCF delta catenin family member	421
TANGO2	transport and golgi organization 2 homolog	128989
TRMT2A	tRNA methyltransferase 2 homolog A	27037
RANBP1	RAN binding protein 1	5902
CCDC188	coiled-coil domain containing 188	388849
DGCR8	DGCR8 microprocessor complex subunit	54487
ZDHHC8	zinc finger DHHC-type palmitoyltransferase 8	29801
RTN4R	reticulon 4 receptor	65078
DGCR6L	DiGeorge syndrome critical region gene 6 like	85359
AC007326.13	novel protein	

B

Category	ID	Name	Source	p-value	q-value Bonferroni	q-value FDR B&Y	Gene List
**Molecular Function**	No results to display						
**Biological Process**							
**Cellular Component**							
**Domain**	IPR010849	Gonadal	InterPro	7.43E-06	1.39E-03	4.04E-03	*DGCR6, DGCR6L*
	PF07324	DGCR6	Pfam	7.43E-06	1.39E-03	4.04E-03	*DGCR6, DGCR6L*
**Pathway**	M39787	22q11.2 copy number variation syndrome	MSigDB C2 BIOCARTA (v7.3)	1.28E-60	2.45E-58	1.43E-57	*TSSK2, RTL10, DGCR2, RANBP1, MRPL40, C22orf39, CLDN5, DGCR6, DGCR8, CLTCL1, TXNRD2, ESS2, TANGO2, TRMT2A, COMT, SEPTIN5, ARVCF, ZDHHC8, DGCR6L, GSC2, SLC25A1, TBX1, CCDC188, RTN4R, GNB1L, PRODH, UFD1, HIRA, GP1BB, CDC45*

Figure S2. Human genes in the proximal deletion of 1.5-Mb on the 22q11.2 locus.
A. Human genes in the proximal deletion of 1.5-Mb on the 22q11.2 locus.
B. Classification of human genes in the proximal deletion of 1.5-Mb. Gene set enrichment analysis was performed using the ToppGene Suite (https://toppgene.cchmc.org) with a Bonferroni corrected q-value cutoff of 0.05.
